# Variation in protection of four divergent avian influenza virus vaccine seed strains against eight clade 2.2.1 and 2.2.1.1. Egyptian H5N1 high pathogenicity variants in poultry

**DOI:** 10.1111/irv.12290

**Published:** 2014-10-03

**Authors:** Erica Spackman, David E Swayne, Mary J Pantin-Jackwood, Xiu-Feng Wan, Mia K Torchetti, Mohammad Hassan, David L Suarez, Mariana Sá e Silva

**Affiliations:** a Southeast Poultry Research Laboratory, USDA-ARSAthens, GA, USA; b Department of Basic Sciences, College of Veterinary Medicine, Mississippi State UniversityMississippi State, MS, USA; c Food and Agriculture Organization of the United Nations, Emergency Centre for Transboundary Animal Diseases, World Organisation for Animal Health and Food and Agriculture Organization of the United Nations Global Network of Expertise on Animal Influenza (OFFLU)Paris, France; d Food and Agriculture Organization of the United Nations, Emergency Centre for Transboundary Animal Diseases, World Organisation for Animal Health and Food and Agriculture Organization of the United Nations Global Network of Expertise on Animal Influenza (OFFLU)Rome, Italy; e National Veterinary Services Laboratories, OFFLUAmes, IA, USA; f National Laboratory for Veterinary Quality Control on Poultry Production, Animal Health Research InstituteDokki, Giza, Egypt

**Keywords:** H5N1, highly pathogenic avian influenza virus, poultry vaccination

## Abstract

**Background:**

Highly pathogenic (HP) H5N1 avian influenza virus (AIV) was introduced to Egyptian poultry in 2006 and has since become enzootic. Vaccination has been utilized as a control tool combined with other control methods, but for a variety of reasons, the disease has not been eradicated. In 2007, an antigenically divergent hemagglutinin subclade, 2.2.1.1, emerged from the original clade 2.2.1 viruses.

**Objectives:**

The objective was to evaluate four diverse AIV isolates for use as vaccines in chickens, including two commercial vaccines and two additional contemporary isolates, against challenge with numerous clade 2.2.1 and clade 2.2.1.1 H5N1 HPAIV Egyptian isolates to assess the variation in protection among different vaccine and challenge virus combinations.

**Methods:**

Vaccination-challenge studies with four vaccines and up to eight challenge strains with each vaccine for a total of 25 vaccination-challenge groups were conducted with chickens. An additional eight groups served as sham-vaccinated controls. Mortality, mean death time, morbidity, virus, and pre-challenge antibodies were evaluated as metrics of protection. Hemagglutination inhibition data were used to visualize the antigenic relatedness of the isolates.

**Results and conclusions:**

Although all but one vaccine-challenge virus combination significantly reduced shed and mortality as compared to sham vaccinates, there were differences in protection among the vaccines relative to one another based on challenge virus. This emphasizes the difficulty in vaccinating against diverse, evolving virus populations, and the importance of selecting optimal vaccine seed strains for successful HPAIV control.

*Please cite this paper as:* Spackman *et al*. (2014) Variation in protection of four divergent avian influenza virus vaccine seed strains against eight clade 2.2.1 and 2.2.1.1: Egyptian H5N1 high pathogenicity variants in poultry. Influenza and Other Respiratory Viruses 8(6), 654–662.

## Introduction

Use of vaccination for the control of avian influenza virus (AIV) has become routine, combined with other control measures such as improved biosecurity in areas where H5N1 highly pathogenic (HP) AIV is endemic in poultry. Historically, only a few H5 AIV seed strains have been used in commercial vaccines. Two of the most widely used have been A/goose/Guangdong/1/1996 which is the source of hemagglutinin (HA) and neuraminidase (NA) genes in the inactivated Re-1 vaccine^1^ (use discontinued in China in mid-2008^2^ although its use continued in Egypt and Vietnam) and A/Mexico/232/1994 H5N2 (Mex/94) low pathogenic (LP) AIV which is used in several inactivated commercial products. Currently, vaccines with the Mex/94 lineage virus and a reverse genetics produced vaccine with an Egyptian 2010 strain antigen are among the vaccines being used. Because of the ability of influenza A viruses to mutate and drift antigenically, there are reports of H5 field viruses which escaped immunity induced by these common vaccines in areas where they have been used widely,^3^–^5^ including Egypt.

H5N1 HPAIV was first detected in poultry in Egypt in 2006.^6^ As then vaccination has been one of the control measures utilized to help control the virus and reduce the impact of infection on poultry. However, H5N1 HPAIV outbreaks have continued to occur and vaccination alone has not been as effective as expected. One of the critical factors known to affect vaccine efficacy is antigenic matching between the challenge virus and vaccine. In countries that have used the same vaccines for an extended period of time, the field viruses have evolved to evade the immune response to the vaccine.^4^,^5^,^7^ Improper vaccination^8^ and inadequate coverage of poultry populations^9^ make control of virus spread difficult which may contribute to the emergence of antigenic variants. In Egypt, a new variant, 2.2.1.1, (previously referred to as ‘variant clade 2.2.1’, ‘sublineage B’ or ‘sublineage E’) first described in 2007 is possibly an example of this.^6^,^10^ The emergence of this clade could complicate vaccination strategies if the currently available vaccines have reduced efficacy against viruses in this clade.

Goals of this work include evaluating the efficacy of the current vaccines against numerous Egyptian isolates including isolates clade 2.2.1.1 and to determine if local, contemporary isolates from Egypt would provide any improvement in protection. Also, as experimental evaluations of AIV vaccines are often published later than is practical, a broader goal was to assess the consistency of protection against multiple challenge viruses, some of which were relatively closely related genetically and antigenically. To accomplish this, we have conducted vaccination-challenge studies with four vaccines and up to eight H5N1 HPAIV challenge viruses per vaccine.

## Materials and methods

### Virus source and propagation

Virus isolates used for vaccine-challenge studies and antigenic cartography were obtained from either the repository at Southeast Poultry Research Laboratory, USDA-ARS (SEPRL; Athens, GA, USA), or the National Laboratory for Quality Control in Poultry Production (NLQP; Cairo, Egypt). All influenza isolates were propagated in specific pathogen-free (SPF) embryonating chicken eggs (ECE) by standard procedures.^11^ Allantoic fluid containing infectious virus was used as challenge virus, and as the antigen source for vaccines and hemagglutination inhibition (HI) assays. The sequence data for the HA genes of all viruses used here have been published with analysis (isolates from 2006 to 2008)^6^ or have been submitted to GenBank (isolates from 2009 to 2010).

### Vaccines

Four viruses were selected for use as vaccine antigens. Two were selected because of their widespread use as commercial vaccines: reverse genetic produced A/goose/Guangdong/1996 H5N1 (GS/GD/96) HPAIV and wild-type A/chicken/Mexico/232/1994 H5N2 (Mex/94) LPAIV. The other two, A/chicken/Egypt/13-NLQP/2008 (Egy/13/08) HPAIV, and A/chicken/Egypt/202-NLQP/2007 (Egy/202/07) HPAIV were selected to represent clades 2.2.1 and 2.2.1.1, respectively.

All vaccines were inactivated, whole virus, adjuvanted in an oil emulsion. The vaccine prepared with GS/GD/96 was the commercial product, Re-1 (Harbin Veterinary Research Institute, Harbin, China),^2^ all others were experimental vaccines prepared in-house as described by Stone *et al*.^12^ using 0·1% beta-propriolactone inactivated allantoic fluid from ECE and adjusting the pH to 7·0 with sodium bicarbonate. Sham vaccine was prepared with allantoic fluid from uninfected ECE. Viruses used to produce vaccines in-house were evaluated for HA titer by standard methods^11^ and by titration in ECE as described previously.^11^ The HA and 50% egg infectious dose (EID_50_) titers for the viruses are given in Table 1.

**Table 1:** Titers of viruses used to prepare vaccines and the geometric mean titer (GMT) of antibody for all birds receiving each vaccine at the time of challenge (3 weeks post-vaccination) based on HI assay

Virus isolate in vaccine	HA titer	EID50 titer (log_10_/ml)	GMT of antibody prior to challenge
A/chicken/Mexico/232/94	512	8·9	49·2^a^
A/chicken/Egypt/202-NLQP/2007	256	8·1	13·6^b^
A/chicken/Egypt/13-NLQP/2008	128	8·7	19·5^b^
A/goose/Guangdong/1996 (Re-1)	Unknown: commercial vaccine		60·6^a^

Superscripts denote statistical groups within the same column.

### Challenge viruses

Eight challenge viruses were selected from among H5N1 HPAIV viruses from poultry in Egypt, aiming to represent a diverse sampling of HA1 protein sequences. The clade 2.2.1 isolates were: A/chicken/Egypt/959-NLQP/2006 (Egy/959/06), Egy/13/08) A/goose/Egypt/20-NLQP/2009 (Egy/20/09), A/duck/Egypt/44-NLQP/2009 (Egy/44/09), A/chicken/Egypt/102d-NLQP/2010 (Egy/2d/10). The clade 2.2.1.1 challenge viruses were as follows: Egy/202/07, A/chicken/Egypt/65-NLQP/2008 (Egy/65/08), and A/chicken/Egypt/1063-NLQP/2010 (Egy/63/10). All viruses were propagated in ECE as described above.

### Vaccination-challenge studies in chickens

Each vaccine-challenge group contained 10 individually tagged, SPF white leghorn chickens from SEPRL in-house flocks. Vaccine-challenge virus groups are listed in Table 2. Chickens were vaccinated between 3 and 4 weeks of age with 0·5 ml of vaccine by the subcutaneous route. Three weeks after vaccination, the birds were bled for serum to evaluate antibody to both the vaccine and challenge virus by HI assay. The birds were also challenged 3 weeks post-vaccination with 10^6^ EID_50_ of HPAIV per bird administered in 0·1 ml by the intrachoanal route.

**Table 2:** Mortality, mean death time, and number of chickens shedding virus at detectable levels in oropharyngeal swabs 2 days post-challenge (DPC) by challenge group

Vaccine	Vaccine HA clade	Challenge virus HA clade	Challenge virus	Percent mortality	Mean death time	Number of chickens orally shedding 2DPC*
Sham	Not applicable	2.2.1	A/chicken/Egypt/959-NLQP/2006	100^c^	2·3^ab^	10/10 (100)^a^
			A/chicken/Egypt/13-NLQP/2008	100^c^	2·4^ab^	4/4 (100)
			A/goose/Egypt/20-NLQP/2009	100^c^	3·2^a^	10/10 (100)^a^
			A/duck/Egypt/44-NLQP/2009	100^c^	2·1^b^	2/2 (100)
			A/chicken/Egypt/2d -NLQP/2010	100^c^	2·7^ab^	10/10 (100)^a^
		2.2.1.1	A/chicken/Egypt/202-NLQP/2007	100^c^	2·1^b^	1/1 (100)
			A/chicken/Egypt/65-NLQP/2008	100^c^	2·6^ab^	10/10 (100)^a^
			A/chicken/Egypt/63-NLQP/2010	100^c^	2·3^ab^	10/10 (100)^a^
A/chicken/Mexico/232/1994 H5N2	Classical H5	2.2.1	A/chicken/Egypt/959-NLQP/2006	0^a^	–	8/10 (80)^a^
			A/goose/Egypt/20-NLQP/2009	0^a^	–	6/10 (60)^a^
			A/chicken/Egypt/2d-NLQP/2010	0^a^	–	7/10 (70)^a^
		2.2.1.1	A/chicken/Egypt/65-NLQP/2008	10^a^	9·0	10/10 (100)^a^
			A/chicken/Egypt/63-NLQP/2010	10^a^	8·0	10/10 (100)^a^
Re-1 Goose/Guangdong/1996 H5N1	0	2.2.1	A/chicken/Egypt/959-NLQP/2006	0^a^	–	8/10 (80)^ab^
			A/chicken/Egypt/13-NLQP/2008	10^a^	3·0	10/10 (100)^a^
			A/goose/Egypt/20-NLQP/2009	0^a^	–	4/10 (40)^b^
			A/chicken/Egypt/2d-NLQP/2010	0^a^	–	7/10 (70)^ab^
			A/duck/Egypt/44-NLQP/2009	0^a^	–	8/10 (80)^ab^
		2.2.1.1	A/chicken/Egypt/202-NLQP/2007	20^ab^	6·0	9/10 (100)^a^
			A/chicken/Egypt/65-NLQP/2008	50^b^	7·0	9/10 (90)^a^
			A/chicken/Egypt/63-NLQP/2010	40^ab^	7·0	10/10 (100)^a^
A/chicken/Egypt/13-NLQP/2008 H5N1	2.2.1	2.2.1	A/chicken/Egypt/959-NLQP/2006	0^a^	–	9/9 (100)^a^
			A/chicken/Egypt/13-NLQP/2008	0^a^	–	9/10 (90)^a^
			A/goose/Egypt/20-NLQP/2009	0^a^	–	2/10 (20)^b^
			A/duck/Egypt/44-NLQP/2009	10^a^	6·0	10/10 (100)^a^
		2.2.1.1	A/chicken/Egypt/202-NLQP/2007	20^ab^	4·0	9/9 (100)^a^
			A/chicken/Egypt/65-NLQP/2008	0^a^	–	7/8 (87·5)^a^
A/chicken/Egypt/202-NLQP/2007 H5N1	2.2.1.1	2.2.1	A/chicken/Egypt/959-NLQP/2006	0^a^	–	8/8 (100)^a^
			A/chicken/Egypt/13-NLQP/2008	30^ab^	2·7	9/10 (90)^a^
			A/goose/Egypt/20-NLQP/2009	0^a^	–	1/9 (11·1)^b^
			A/duck/Egypt/44-NLQP/2009	70^bc^	6·1	10/10 (100)^a^
		2.2.1.1	A/chicken/Egypt/202-NLQP/2007	20^ab^	3·5	10/10 (100)^a^
			A/chicken/Egypt/65-NLQP/2008	0^a^	–	7/8 (87·5)^a^

Superscript letters denote the statistical group within the same column (groups without superscripts were not included in statistical calculations due to inadequate sample numbers).

* Number positive/total (% positive).

Oropharyngeal swabs were collected 2 days post-challenge (DPC) to evaluate virus shed titers by quantitative real-time RT-PCR (qrRT-PCR).

### Quantitative real-time RT-PCR

Oropharyngeal swabs collected at 2 DPC were processed for qrRT-PCR to determine virus shed titers. RNA was extracted as described by Das *et al*.^13^ using a combination of Trizol LS (Invitrogen, Inc., Carlsbad, CA, USA) and the MagMAX 96 AI/ND Viral RNA isolation kit (Ambion, Inc., Austin, TX, USA) with the KingFisher magnetic particle processor (Thermo Scientific, Waltham, MA, USA).

Quantitative rRT-PCR which targets the influenza M gene^14^ was performed using the 7500 FAST Real-time PCR System (Applied Biosystems, Foster City, CA, USA) and the AgPath-ID OneStep RT-PCR kit (Ambion, Inc.) in accordance with the U.S. National Veterinary Services Laboratories protocol AVSOP1521.01. The standard curve for virus quantification was established with RNA extracted from dilutions of the same titrated stock of each virus used to challenge the chickens and was run in duplicate.

### Hemagglutination inhibition assay

Sera were tested by the HI assay to evaluate antibody levels to both the vaccine and challenge virus in vaccinated birds pre-challenge. Because GS/GD/96, the source of the HA in the Re-1 vaccine was not available, a closely related isolate which was available was used: A/HongKong/156/1997 H5N1 (HK/156) (98·1% amino acid identity in the HA1).

The assay antigens were prepared by inactivating infectious allantoic fluid with 0·1% beta-propiolactone and adjusting the pH to 7·0 with sodium bicarbonate. The HI assays were performed in accordance with standard procedures.^15^ Titers were calculated as the reciprocal of the last HI positive serum dilution.

### Antigenic cartography

A total of 23 H5N1 HPAIVs from Egypt were selected for characterization by antigenic cartography. Each virus used as a vaccine and/or challenge strain was included. Additional isolates were selected to represent divergent H5 HA1 protein sequences. In addition to the isolates from Egypt, five H5N1 isolates from different HA clades were included (the HK/156 antigen was used to approximate GS/GD/96). Two classical H5 clade viruses were also used, one of which was the Mex/94 isolate included as one of the vaccines in this study.

Reference sera were produced with an inactivated, oil emulsion vaccine prepared with the Montanide ISA50V adjuvant (Seppic, Inc., Fairfield, NJ, USA) using allantoic fluid prepared as described under HI assay. Three-week-old SPF white leghorn chickens from SEPRL in-house flocks were inoculated with 0·5 ml of vaccine by the subcutaneous route, and serum was collected 3 weeks post-vaccination. Sera were treated with 5% chicken red blood cells for 30 minutes at ambient temperature to adsorb non-specific agglutinins. The HI assay was performed by standard procedures.^15^

The antigenic map for the test antigens was constructed using matrix completion-multiple dimensional scaling (MC-MDS).^16^ Given the HI table h with *n* antigens (*n *=* *30 in this study) and *m* antisera (*m *=* *28 in this study), each observed value *h*_*ij*_ was transformed into 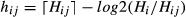, where *H*_*ij*_ is the maximum HI titer in the HI table h and *H*_*j*_ is the maximum HI titer for serum *j,* 1 ≤ *i *≤ *n*, and 1 ≤ *j *≤ *m*. The value of 2 was defined as a low reactor, and a value of 4096 was used for those entries with ‘>4096’. A low rank matrix completion algorithm was applied to identify those entries without experimental data and to reduce the noise in HI table. The antigenic distance was then calculated and mapped to a two dimensional antigenic map.

### Statistical methods

Shed titers by vaccine and challenge virus, mean death times, and antibody titers to the vaccine and challenge virus prior to challenge were tested for statistical significance using the one-way anova, all pairwise (Tukey test). If normality failed then the Student–Newman–Keuls method was used (pairwise). Linear regression was used to evaluate the correlation between antibody levels to the vaccine or challenge virus and virus shed titers. All statistical analyses were conducted with sigmaplot 11.2 (Systat Software, Richmond, CA, USA). A *P* value of <0·05 was considered to be significant. Statistical groups are denoted in tables as superscript and on graphs by lowercase letters. Groups with different letters are significantly different at a *P* value of <0·05. Groups apply to values in the same column. Samples which were negative by rRT-PCR were counted as below the limit of detection (titer = 10 EID 50/ml).

## Results

### Serology

**Figure 1:**
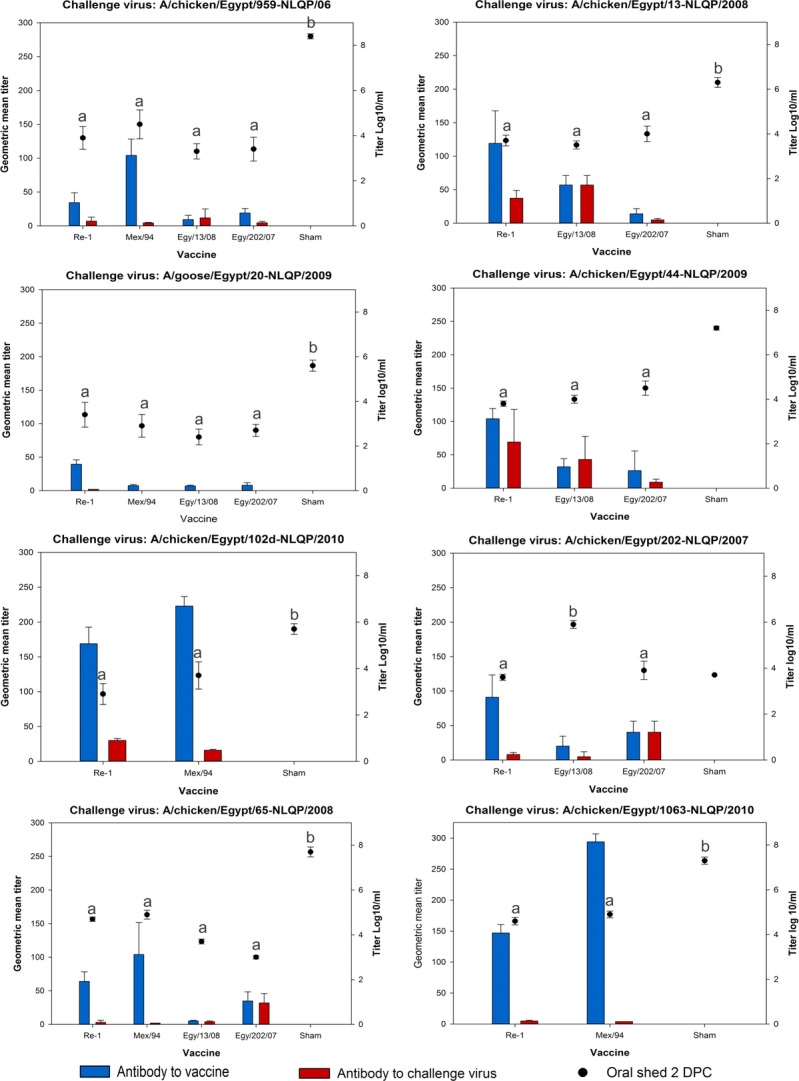
Pre-challenge geometric mean antibody (Ab) titers (determined by HI assay) to both the vaccine virus and challenge virus versus oral shed titers (log_10_/ml) 2 days post-challenge. Statistical groups (*P* < 0·05) for mean shed titers are denoted by a letter where different letters indicate statistical difference (mean shed titers without a letter indicate insufficient surviving birds to compare shed titers). Error bars represent standard error.

Antibody levels to each vaccine 3 weeks post-vaccination (immediately prior to challenge) were evaluated by HI assay (Figure 1). The Re-1 vaccine (commercial product) and the in-house vaccine produced with the Mex/94 isolate produced significantly higher levels of antibody (geometric mean titers) than either of the experimental vaccines produced with the Egyptian H5N1 isolates: Egy/202/07 or Egy/13/08. Antibody levels using the challenge viruses as test antigen in the HI assay for each group varied widely by vaccine-challenge virus combination, but were consistently lower than antibody levels to the vaccine (in all non-homologous groups) (Figure 1).

Amount of virus shed was evaluated against antibody titer to either the vaccine or challenge virus immediately prior to challenge. There was no correlation between the level of virus shed and the antibody titer to either the vaccine or challenge virus for any of the treatment groups. There was also no association between antibody titers and mortality.

### Vaccination challenge

Four vaccines were evaluated with up to eight H5N1 challenge viruses from Egypt for a total of 25 vaccine-challenge virus combinations and eight additional sham vaccinated groups (Table 2). Protection was measured as prevention of mortality and decrease in virus shed. There was 100% mortality in all sham vaccinated groups and mortality varied by vaccine-challenge virus combination (Table 2). There was not a clear trend for prevention of mortality in groups vaccinated with Egy/202/07 or Egy/13/08 based on challenge virus clade; however, in groups vaccinated with Re-1 or Mex/94 mortalities were nearly exclusive to the groups challenged with viruses from clade 2.2.1.1. This trend was more pronounced with the Re-1 vaccinated group. When collectively evaluated by vaccine and clade (mortality from all challenge viruses of the same clade combined) mortality was significantly higher for the chickens challenged with clade 2.2.1.1 isolates versus clade 2.2.1 isolates with Re-1, Mex/94 and Egy/13/08 vaccinated groups and significantly lower in the Egy/202/07 vaccinated groups.

Oral virus shed at 2 DPC varied by vaccine and challenge virus combination (Figure 1); all vaccines significantly reduced shed compared to sham vaccinated chickens except for the group vaccinated with Egy/202/07 and challenged with Egy/13/08 (note that the groups challenged with Egy/202/07, Egy/44/09 could not be statistically evaluated versus sham vaccinates, because there was only one or two sham vaccinates, respectively, which survived long enough to be sampled for shed). All vaccines reduced shed similarly for seven of the eight challenge viruses; that is, the titers of virus shed was not significantly different among vaccinated birds for any virus, regardless of vaccine (Figure 1).

**Figure 2:**
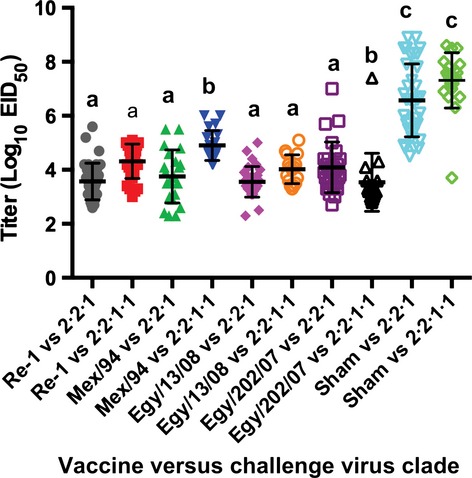
Oral virus shed 2 day post-challenge collectively by HA clade of challenge virus and vaccine. Letter denote statistical groups (*P* < 0·05) where different letters indicate statistical difference. Error bars represent standard deviation.

When virus shed was compared between the two clades for each vaccine, the differences in mean titers were significantly lower with challenge viruses from clade 2.2.1 versus challenge viruses from clade 2.2.1.1 for Re-1, Mex/94, and Egy/13/08 (Figure 2). In contrast, shed titers were significantly higher with challenge viruses from clade 2.2.1 than clade 2.2.1.1 from chickens vaccinated with Egy/202/07.

### Antigenic profiling by antigenic cartography

**Figure 3:**
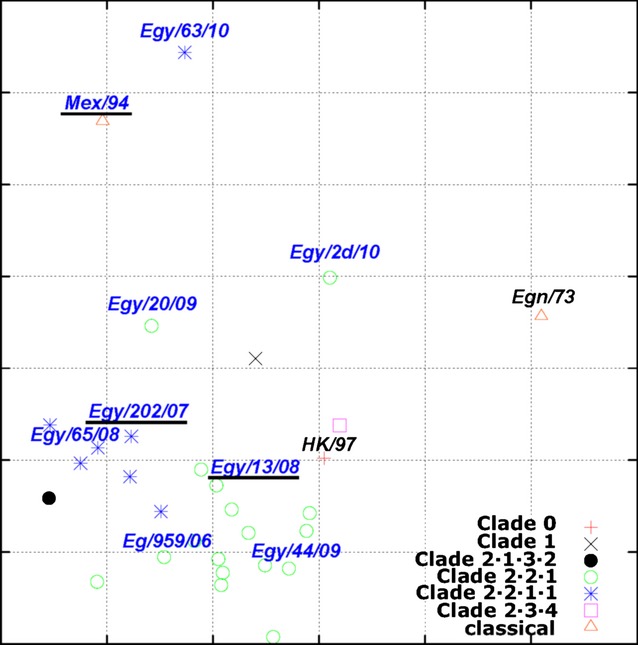
Influenza antigenic cartography for the vaccine strains and Egyptian H5N1 HPAIVs. Both the vaccine strain and challenges are labeled in blue text, and the vaccine strains are underlined. The antigenic cartography is constructed using AntigenMap (http://sysbio.cvm.msstate.edu/AntigenMap).

Antigenic mapping showed that the Egyptian H5N1 viruses in clade 2.2.1 do predominantly cluster apart from the Egyptian H5N1 viruses in clade 2.2.1.1 (Figure 3). The antigenic distances within clade 2.2.1 is 1·4609 ± 0·7470 (mean ± standard deviation) antigenic units (an antigenic unit is equal to a twofold difference in HI assay titer), and within clade 2.2.1.1 is 1·6291 ± 1·3042 units. However, the intercluster distance is 2·1875 ± 0·9904 units. The antigenic distances within each clade (2.2.1 or 2.2.1.1) are statistically smaller than those between these two clades, *P* < 0·001 (this result is consistent to those reported previously^17^). The antigenic distances between the vaccine strains and challenge viruses are shown in Table 3.

**Table 3:** Antigenic distances in antigenic units of the vaccine strains and challenge viruses used in this study

Virus	Clade	Abbreviation	Virus									
			Classical	0	2.2.1					2.2.1.1		
			Mex/94	HK/97	Eg/959/06	Egy/13/08	Egy/20/09	Egy/44/09	Egy/2d/10	Eg/202/07	Egy/65/08	Egy/63/10
A/chicken/Hidalgo/232/1994*	Classical	Mex/94	0·0000	3·6026**	3·9055	3·1541	2·5579	4·1733	2·7647	3·0629	3·1768	1·5096
A/Hong Kong/156xPR8/1997***	0	HK/97		0·0000	1·9033	1·4016	1·9132	1·4025	2·5845	2·2506	2·5004	3·9931
A/chicken/Egypt/959-NLQP/2006	2.2.1	Eg/959/06			0·0000	1·0912	2·3717	1·4155	3·0723	1·4924	1·6606	4·4884
**A/chicken/Egypt/13-NLQP/2008**	2.2.1	Egy/13/08				0·0000	1·8489	1·4559	2·1018	1·0159	1·2712	3·5764
A/goose/Egypt/20-NLQP/2009	2.2.1	Egy/20/09					0·0000	2·3698	2·6067	2·0364	2·2999	3·0164
A/duck/Egypt/44-NLQP/2009	2.2.1	Egy/44/09						0·0000	2·7450	2·1748	2·3811	4·7060
A/chicken/Egypt/102d-NLQP/2010	2.2.1	Egy/2d/10							0·0000	2·1433	2·2167	2·7389
**A/chicken/Egypt/202-NLQP/2007**	2.2.1.1	Egy/202/07								0·0000	0·3058	3·3080
A/chicken/Egypt/65-NLQP/2008	2.2.1.1	Egy/65/08									0·0000	3·3692
A/chicken/Egypt/1063-NLQP/2010	2.2.1.1	Egy/63/10										0·0000

* The viruses in bold were used as vaccines.

** One antigenic unit is equal to a twofold difference in HI.

*** HK/97 strain is used as an antigenically similar strain to GS/GD/96 from clade 0.

## Discussion

As expected, minor differences in protection (reduction of mortality and reduction of virus shed) based on vaccine and challenge virus combination were observed. Also there were higher mortality and shed titers in the Re-1 vaccinated groups that were challenged with clade 2.2.1.1 isolates versus clade 2.2.1 isolates. This data support the hypothesis that antigenic drift of H5N1 HPAIV in Egypt may be a result of immune pressure, and highlights the need for updated vaccines. In mid-2008, Re-1 was discontinued in China^2^ where was replaced by a vaccine with a more recent H5N1HA gene, although, use of Re-1 continued for at least another year in Egypt. Continued use of Mex/94 based vaccines should also be critically reviewed; collective analysis of the two clade 2.2.1.1 challenge isolates tested here showed significantly more virus shed versus the clade 2.2.1 challenge isolates, although mortality was not significantly different. The protection provided by Mex/94 will likely continue to deteriorate from its current levels similar to what has been noted with long term use of AIV vaccines in poultry in the past.^3^–^5^

Our results are similar to what was reported by Grund *et al*.^18^ based on protection by clade, although they used different challenge viruses from Egypt (one from each clade) and three of their four vaccines were different (Mex/94 was used commonly). Grund *et al*.'s study also showed that the best protection against mortality and reduction of shed against clade 2.2.1.1 challenge was by vaccination with a clade 2.2.1.1 virus. While improved protection was noted with the clade 2.2.1.1 based vaccine in this study, it was still poorer than the protection Re-1 and Mex/94 provided against the clade 2.2.1 viruses.

Although there are differences among the vaccines in overall reduction of virus shed based on challenge virus clade, individually 17 of 18 vaccinated groups (the seven groups challenged with either Egy/202/07 or Egy/44/09 were not included in the statistical analysis because too few sham vaccinates survived long enough to be tested to determine the mean shed titer with adequate power) did reduce shed significantly versus sham vaccinates. Individually, 24 of 25 of the vaccine challenge groups also had significantly lower mortality when compared to sham vaccinates challenged with the same isolate. This has important implications for evaluating vaccines because most vaccines will provide measurable protection against mortality when compared to non-vaccinated or sham vaccinated birds in an experimental setting, but optimal protection may only be recognized in comparison to less protective vaccines. Furthermore, protection is always better in the lab than in the field; small differences in an experimental setting may be amplified in the field.

Somewhat interestingly, there was no significant difference in the number of chickens shedding detectable levels of virus based on vaccine or challenge virus clade. There was one challenge virus, Egy/20/09, for which significantly fewer chickens shed virus with three of the four vaccines (the exception was Mex/94). This virus also had the longest MDT with the shams, although this was only significant as compared to two other isolates. This suggests that vaccine efficacy may differ even among relatively closely related challenge viruses due to minor biological variations; if this isolate had been the only one used most of the vaccines would have appeared to have been more efficacious at reducing shed than they were. Because halting the transmission is a primary goal of vaccination, the optimal vaccines will substantially reduce both the shed titers and number of animals shedding virus. Interestingly, through 2012 the 2.2.1 clade remained predominant; the 2.2.1.1 clade did not replace it,^19^ reinforcing that the selection pressures on influenza are numerous (e.g., immune pressure, biological, epidemiological), and complex.

The serological results were also interesting in that antibody titers to neither the vaccine nor the challenge virus were predictive of either survival or shed. This is in contrast to the somewhat loosely held principle that HI titers over 40 indicate that a bird will be protected from mortality^20^ and greater than 120 prevent replication.^21^ However, others have reported good protection against mortality in groups when geometric mean titers were below an HI titer of 40^22^–^24^ and it has been recognized that this cut-off is less predictive with vaccine viruses which are not well matched to the challenge virus among other factors.^25^ This may be because HI assay is not measuring antibody to all protective epitopes, such as antibody to the fusion domain of the HA protein^26^ or some other immunological component induced by inactivated adjuvanted vaccines.

Serology also revealed that Re-1 and Mex/94 induced higher antibody levels than either of the Egyptian isolates. Importantly, the differences in antibody levels did not translate into differences in protection. The lower immunogenicity of the Egyptian isolates is probably not due to vaccine formulation since the Mex/94 vaccine was prepared in-house identically to the Egyptian virus based vaccines and antigenic masses were similar based on HA titer. Therefore, there may be an inherent difference in the immunogenicity of the HA proteins of these isolates for chickens. Poor immunogenicity may be under selection to evade the immune response and may help explain why there was mortality with Egy/202/07 homologous vaccination challenge. Poorly immunogenic HA protein is thought to be why vaccines produced with A/chicken/Pakistan/NARC-1/1995 H7N3 produced poor immunity in the field and protected poorly against homologous challenge in experimental studies.^27^,^28^ Conversely, good immunogenicity may explain why some isolates can provide excellent protection against relatively unrelated challenge viruses (e.g., Re-1 and Mex/94 vaccines with clade 2.2.1 challenge viruses).^29^ As antigenic proximity did not correlate with protection based on the HI assay data, these data suggest that immunogenicity may be at least as critical as antigenic matching when selecting vaccine seed strains. Importantly, the observed segregation based on clade has been previously reported.^17^ With this data HI based antigenic profiling alone is not sufficient to draw more general conclusions and nor is it clear what the effect of adjuvant may be on antibody reactivity. Finally, when updating vaccines, protection needs to be evaluated carefully, as simply using a recent local virus of the same genetic lineage may not always provide optimal protection.
